# Design, synthesis and neuroprotective activity of compound derived from *Gastrodia elata* Blume and borneol

**DOI:** 10.3389/fphar.2024.1437806

**Published:** 2024-09-23

**Authors:** Lijuan Zhi, Huan Li, Baimei Shi, Tao Yu, Xiaoni Jia, Hui Zhang

**Affiliations:** ^1^ Center Laboratory, Xi’an Mental Health Center, Xi’an, China; ^2^ College of Chemical Engineering, Xi’an University, Xi’an, China

**Keywords:** *Gastrodia elata* Blume, borneol, neuroprotection, glutamate, cerebral ischemia

## Abstract

**Introduction:**

Traditional Chinese medicine *Gastrodia elata* Blume (GEB) possesses properties that soothe the liver and dispel wind. Its constituents exhibit numerous pharmacological properties, including neuroprotective effects, analgesic properties for headache relief, memory enhancement, and others. Borneol enhances drug absorption by traversing the blood-brain barrier, thereby improving its bioavailability and therapeutic efficacy. The research aimed to design innovative drug molecules and contribute to the beneficial exploration of compound Chinese medicine modernization.

**Methods:**

This study employed the strategy of “compound Chinese medicine molecular chemistry” to integrate and fuse the effective substances of compound Chinese medicines. An excitotoxic injury model was established by exposing PC12 cells to glutamate. Cell viability was quantitatively evaluated utilizing a colorimetric assay with the CCK-8 reagent kit. Genecards, Disgenet, and OMIM databases were used to identify potential disease-related targets. Molecular docking methods were performed to predict the binding interactions between compounds and core targets.

**Results:**

We designed and synthesized compounds TB-1 to TB-16. Following the evaluation of their safety, TB-1, TB-2, TB-12, and TB-16 were selected for further investigation of their neuroprotective properties. The compound designed in this study exhibits a dose-dependent protective effect on glutamate-damaged PC12 cells. Further network pharmacology and molecular docking analyses indicate that TB-2 possesses a potential therapeutic effect against cerebral ischemia, and its possible targets were SRC, MAPK1 and KDR.

**Discussion:**

The results indicated that TB-2 displayed a significant neuroprotective effect against Glu-induced injury in PC12 cells, suggesting potential therapeutic implications for cerebral ischemia.

## 1 Introduction

The Chinese medicinal formula, *Longnao Tianma Jian*, derived from the *Taiping Huimin Heji Ju Fang*, exhibits the efficacy of cooling blood, quenching wind, alleviating dysmenorrhea, and activating collaterals, thereby addressing conditions associated with wind disorders and slow wind paralysis (Liu, 2007).Within this prescription, *Gastrodia elata* Blume (GEB) and borneol are two pivotal ingredients. GEB, also known as Tianma, possesses significant clinical application value as a traditional Chinese herbal medicine. GEB exhibits therapeutic potential in treating headaches, strokes, amnesia, and various other conditions (Sun et al., 2023). The therapeutic potential of the primary components of GEB for nervous system disorders has garnered increasing attention. 4-hydroxybenzaldehyde, isolated from GEB, exhibits neuroregulatory activity and efficiently mitigates neuronal damage resulting from cerebral ischemia (Xiao et al., 2022; Yu et al., 2023).

Borneol, a bicyclic terpene with high fat solubility, has been demonstrated through pharmacological studies to enhance the bioavailability and therapeutic efficacy of drugs by serving as a “drug carrier” across the blood-brain barrier, thereby facilitating drug absorption (Kulkarni et al., 2021; Zhang et al., 2017). Moreover, borneol prevents neuronal injury following cerebral ischemia via diverse mechanisms, thereby mobilizing endogenous trophic factors to facilitate brain tissue repair and regeneration (Li et al., 2021).

Cerebral ischemia is a condition resulting from neuronal cell death, calcium overload, cholinergic dysfunction, and excitability toxicity, among other mechanisms (Ahad et al., 2020). Although cerebral ischemia is generally regarded as an age-related disease, the age of onset has significantly decreased in recent years, attributed to unhealthy diet, stressful lifestyle, and deteriorating environmental quality (Kaviarasi et al., 2019). Consequently, there is a pressing need for effective therapeutic strategies to mitigate the impairment of brain function.

Glutamate (Glu) is an excitatory neurotransmitter that plays a pivotal role in the central nervous system. Glu can induce neuronal cell death via Glu receptor-mediated excitotoxicity and ROS-mediated oxidative toxicity (Neves et al., 2023). Studies have shown that Glu is implicated in the etiology and pathology of various neurological diseases, including brain trauma (Tehse and Taghibiglou, 2019), stroke (Shen et al., 2022), Alzheimer’s disease (Song et al., 2021), Parkinson’s disease (Iovino et al., 2020) and other diseases. Therefore, investigating the protective effects against glu-induced neurotoxicity has emerged as a therapeutic strategy for the treatment of neurodegenerative diseases. In previous studies, PC12 cells often used to construct the glutamic acid damage model (Chang et al., 2020; Alavi et al., 2023).

In traditional Oriental medicine, it is conventional to combine different herbs to simultaneously achieve a variety of therapeutic purposes or enhance a single effect without causing severe side effects (Li et al., 2022). The molecules are combined or merged using chemical methodologies to form a novel innovative drug design concept (Li et al., 2021). These new synthetic drug molecules can achieve the effect of improving the original drug group. In this paper, the strategy of “compound Chinese medicine molecular chemistry” was employed to integrate. Based on the molecular chemistry of combined traditional Chinese medicine, the active constituents of GEB and borneol were appropriately combined at the pharmacophore level. The neuroprotective effects of these compounds were investigated through the establishment of a PC12 cell model of glutamate injury. Additionally, this research further elucidates the potential targets of neuroprotective compounds in cerebral ischemic diseases. This study aims to contribute to the exploration of the modernization strategies for traditional Chinese medicinal compounds.

## 2 Materials and methods

### 2.1 Materials

*L*-glycine ethyl ester, *L*-isopic acid ethyl ester, and *L*-phenylalanine ethyl ester were purchased from Beijing InnoChem Science and Technology Co., Ltd., while *L*-leucine were obtained from Sinopharm Chemical Reagent Co., Ltd. Additionally, *L*-alanine ethyl ester, *L*-cypacorinine ethyl ester, L-serine ethyl ester, and *L*-tyrosine ethyl ester were procured from Bide Pharmatech Co., Ltd., along with Toto fluoride, EDC·HCl, 1-hydroxybenzotriazole, 4-dimethylaminopyridine, acetohydroxyacid synthase, and ethyl acetate. PC12 cell was purchased from Wuhan Puno Life Science and Technology Co., Ltd., Cell Counting Kit-8 (CCK-8) was acquired from Abbkine Biotechnology Co., Ltd., and Glu was purchased from Xintai Biological Technology Development Co., Ltd. Nimodipine was sourced from Tianjin Heowns Biochem LLC. Trypsin-EDTA solution was obtained from Beijing Solarbio Science and Technology Co., Ltd. Dulbecco’s modified Eagle’s medium (DMEM) was purchased from Thermo Fisher Scientific, and fetal bovine serum (FBS) was procured from Shanghai XP Biomed Ltd. 200–300 mesh silica gel was purchased from Qingdao Haiyang Chemical Co., Ltd. (China).

### 2.2 Procedure for the synthesis of intermediates substituted acetic acid (1a, 2a), and the target molecules (TB-1∼TB-16)

All commercially available reagents were used without further purification. Anhydrous solvents were dried following standard procedures. Flash column chromatography was carried out on 200–300 mesh silica gel. Reactions were monitored via thin-layer chromatography (TLC) on silica gel plates (GF254) and visualized under UV light. ^1^H-NMR and ^13^C-NMR spectra were recorded using a Bruker AV-400 spectrometer (Bruker Company, Germany) in the specified solvents (CDCl_3_, DMSO-d_6_ or CD_3_COCD_3_, with TMS as the internal standard). The chemical shift values are expressed in δ values (ppm), and the coupling constants (J) are reported in Hz. High-resolution mass spectrometry (HRMS) were measured using a Finnigan MAT 95 spectrometer (Finnigan, Germany). Melting points, which are uncorrected, were measured using a digital melting point apparatus (Shenguang WRS-1B, Shanghai, China). The purity of the synthesized compounds, analyzed by HPLC (Waters 2695 Alliance system) using a Kromasil C18 column eluted with a gradient of acetonitrile/water (15/85-95/5) containing 0.1% formic acid at a flow rate of 1 mL/min, was over 95%.

To a solution of 4-hydroxylphenyl aldehyde (12.2 g, 0.10 mol) and K_2_CO_3_ (20.7 g, 0.15 mol) in anhydrous DMF (150 mL) was added ethyl 2-bromoacetic acid (16.5 g, 0.10 mol) drop wise. After complete addition the mixture was stirred at 50°C for 24 h until 4-hydroxylphenyl aldehyde was consumed. The mixture was poured into ice water to precipitate solid. After filtration and washing with petroleum ether and water several times the obtained solid was yielded. The obtained ester was hydrolyzed in ethanol using sodium hydroxide solution at 50°C for 3 h. The suspending solution was adjusted to pH3-4 value and concentrated to remove some of solution. The residue was cooled to 5°C and the precipitated solid was collected. After being dried at 55°C for 24 h compound **1a** was obtained (15.8 g, 87.8%yield).

The key intermediate **1a** (1.0 g, 5.55 mmol) was activated with thionyl chloride (3 mL, 41.34 mmol) in dichloridemethane. The mixture was heated to reflux and kept for 6 h. After concentrated in reduced pressure the acid chloride was obtained. The acid chloride was added drop wise to a solution of amino acid ester (5.55 mmol) and Et_3_N (1.12 g, 11.1 mmol) in dichloridemethane at 0°C–5°C over 20 min. After complete addition the mixture was stirred for 1 h. The mixture was extracted with dichloridemethane and washed with diluted HCl solution and water. The obtained organic layer was dried and concentrated to yield (4-formylphenoxy) group substituted amino acid ester which was hydrolyzed using sodium hydroxide to give (4-formylphenoxy) group substituted amino acid **2a** (in 64%–84% yield).

To a solution of (4-formylphenoxy) group substituted amino acid **2a** (3.0 mmol) in dichloromethane was added EDC (0.86 g, 4.5 mmol) and DMAP (73.2 mg, 0.6 mmol). After stirred for 10 min borneol (462 mg, 3.0 mmol) was added. The reactant mixture was stirred at room temperature for 24 h until the starting material was consumed completely. The mixture was extracted with dichloromethane, washed with water and brine. The obtained organic layer was dried over anhydrous Na_2_SO_4_ and concentrated in reduced pressure to give crude product. After purification by silica gel column chromatography the pure target molecules (**TB-1∼TB-16**) was yielded in 73%–89% yield.

### 2.3 Cell culture

PC12 cells were cultured in DMEM supplemented with 10% FBS under a humidified, 37°C, 5% CO_2_ atmosphere in a constant-temperature carbon dioxide incubator (Thermo Fisher Scientific, United States of America). The cell state was observed under an inverted fluorescent microscope (Leica, Germany). PC12 cells were cultured and expanded in culture flasks until reaching 90% confluence, whereupon they were used for the experiments.

The cells were counted under a positive microscope (Leica, Germany). For viability assessment, the cells were seeded at a density of 5 × 10^4^ cells/mL in 96-well plates, with 100 µL dispensed into each well. Following adherence to the culture surface, these cells were subsequently utilized for experiments.

TB-1 ∼ TB-16 were dissolved in DMEM medium and prepared as drug-containing media at concentrations of 0.1, 1, 10, 25, 50, 100, and 250 μM. PC12 cells were pre-incubated with TB-1 to TB-16 for 24 h prior to assessing the safety profile of the compounds. Glu powder was dissolved in DMEM to achieve a final concentration of 50 mM. Selected compounds, TB-1, TB-2, TB-12, and TB-16, were pre-applied to PC12 cells at concentrations of 0.1, 1, 10, and 100 μM for 24 h, followed by exposure to Glu treatment (final concentration: 7 mM) for an additional 24 h.

Following drug treatment, cell viability was quantitatively evaluated utilizing a colorimetric assay with the CCK-8 kit, wherein CCK-8 reagent was diluted 1:10 in DMEM to serve as the assay medium. Cells were incubated with CCK-8 reagents for 1 h at 37°C in a dark environment. Subsequently, the absorbance at 450 nm was measured using a Microplate Reader (BioTek, United States of America). The viability of treated cells was expressed as a percentage relative to that of control cells, calculated using the formula: 100 × (OD of Glu-drug-treated − OD of blank control)/(OD of control − OD of blank control). The viability of the untreated group was set as 100%, and each experiment was repeated at least three times.

### 2.4 Molecular modeling study

The Genecards, Disgenet, and OMIM databases were queried using the keyword “cerebral ischemia” to retrieve related disease targets. In the Genecards database, the Score value indicates the proximity of the target to the disease, and the median Score value was used as a screening criterion. After consolidating the targets from the three databases, duplicates were removed. The compound’s structure was imported into the Swiss Target Prediction database to identify potential targets, with a score threshold of greater than 0, indicating the action target of the compound. The identified targets for the chemical compound and cerebral ischemia were simultaneously input into the Venny online platform to determine the common targets. These common targets were then analyzed in the String database, to construct a PPI network of chemical compounds on cerebral ischemia targets. Core targets were screened using Cytoscape 3.9.1 software.

The 3D structures of SRC, MAPK1, KDR, GSK3B, EGFR, and NR3C2 were retrieved from the RCSB Protein Data Bank (PDB). These protein structures were subsequently opened in Autodock 4.2.6, where water molecules were removed and hydrogen atoms added. The structures of TB-2 and TB-12 were optimized and designated as ligands. Subsequently, the prepared ligand and protein structural files were concurrently imported into Autodock 4.2.6 for docking simulations. The software then generated multiple conformations of the ligand bound to the protein and assessed their respective binding energies. These docking results were then imported into PyMOL 2.2.0 for visual representation and analysis.

### 2.5 Statistical analysis

The data were collected from replicated experiments, with outliers exhibiting significant deviations removed to ensure at least three replicates per group. The findings are reported as means ± SEM derived from these replicated measurements. Statistical analysis was performed using one-way ANOVA, implemented within GraphPad Prism 10.0.2 software. Significance was defined as *p*-values <0.05.

## 3 Results

### 3.1 The target molecules analytical data

**FIGURE 1:**
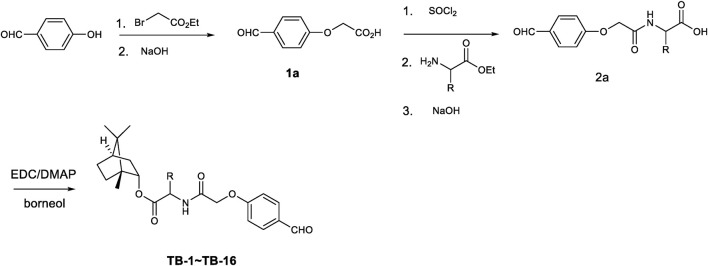
Synthesis of 4-hydroxylphenyl aldehyde and bornel derivatives.

All compounds TB-1∼TB-16 were successfully synthesized using the synthetic protocols outlined in Figure 1. The purity of TB-1∼TB-16 were greater than 95%. The identification of compounds TB-1∼TB-16 is listed as follows.

(1R,2S,4S)-1,7,7-trimethylbicyclo[2.2.1]heptan-2-yl (2-(4-formylphenoxy)acetyl) glycinate (**TB-1**): 78% yield; ^1^H NMR (400 MHz, CDCl_3_) δ 9.90 (s, 1H), 7.88 (s, 1H), 7.04 (s, 1H), 4.98 (d, *J* = 31.7 Hz, 1H), 4.61 (s, 1H), 4.19 (s, 1H), 2.45-2.26 (m, 1H), 1.92-1.63 (m, 2H), 1.26 (dd, *J*_1_ = 21.5 Hz, *J*_2_ = 13.6 Hz, 1H), 1.00 (dd, *J*_1_ = 13.8 Hz, *J*_2_ = 3.4 Hz, 1H), 0.89 (s, 3H), 0.86 (s, 3H), 0.82 (s, 3H); ^13^C NMR (101 MHz, CDCl_3_) δ 190.6, 169.6, 167.4, 161.8, 132.1, 131.1, 115.0, 81.7, 77.4, 77.1, 76.7, 67.2, 53.4, 48.9, 47.9, 44.8, 41.1, 36.6, 27.9, 27.0, 19.6, 18.8, 13.5; HRMS (ESI): Exact mass calcd for C_21_H_27_NO_5_, [M + Na]^+^, 396.1781. Found 396.1785.

(1R,2S,4S)-1,7,7-trimethylbicyclo[2.2.1]heptan-2-yl (2-(4-formylphenoxy)acetyl) alaninate (**TB-2**): 79% yield; ^1^H NMR (400 MHz, CDCl_3_) δ 9.91 (s, 1H), 7.88 (d, *J* = 8.7 Hz, 2H), 7.17 (d, *J* = 6.6 Hz, 1H), 7.07 (d, *J* = 8.7 Hz, 2H), 5.00-4.89 (m, 1H), 4.76-4.65 (m, 1H), 4.59 (d, *J* = 1.3 Hz, 2H), 2.51-2.25 (m, 1H), 1.93-1.83 (m, 1H), 1.81-1.63 (m, 2H), 1.48 (dd, *J* = 7.1 Hz, 3.1, 3H), 1.27 (td, *J*_1_ = 17.6 Hz, *J*_2_ = 10.0 Hz, 3H), 1.02 (dd, *J*_1_ = 13.8, *J*_2_ = 3.4, 1H), 0.90 (s, 3H), 0.88 (s, 3H), 0.83 (s, 1H), 0.82 (s, 2H); ^13^C NMR (101 MHz, CDCl_3_) δ = 190.6, 172.7, 172.6, 166.6, 161.8, 132.1, 131.1, 115.0, 81.6, 81.5, 77.3, 77.03, 76.7, 67.2, 49.0, 48.8, 48.1, 47.9, 47.9, 44.8, 44.8, 36.8, 36.4, 28.0, 27.9, 27.0, 19.6, 18.8, 18.5, 13.50, 13.4; HRMS (ESI): Exact mass calcd for C_22_H_29_NO_5_, [M + Na]^+^, 410.1943. Found 410.1940.

(1R,2S,4S)-1,7,7-trimethylbicyclo[2.2.1]heptan-2-yl (2-(4-formylphenoxy)acetyl) valinate(**TB-3**): 75% yield; ^1^H NMR (400 MHz, CDCl_3_) δ 9.91 (s, 1H), 7.87 (d, *J* = 8.8 Hz, 2H), 7.15-6.91 (m, 3H), 5.00-4.84 (m, 1H), 4.69-4.58 (m, 3H), 2.42-2.05 (m, 3H), 1.95-1.81 (m, 1H), 1.80-1.61 (m, 2H), 1.37-1.17 (m, 3H), 0.96 (m, 7H), 0.89 (s, 3H), 0.87 (s, 3H), 0.82 (s, 3H); ^13^C NMR (101 MHz, CDCl_3_) δ 190.6, 171.6, 167.0, 161.8, 132.1, 131.1, 115.1, 81.7, 77.4, 77.1, 76.7, 67.3, 56.9, 48.8, 47.9, 44.8, 36.7, 31.6, 27.9, 27.2, 19.7, 18.9, 18.8, 17.6, 13.6; HRMS (ESI): Exact mass calcd for C_24_H_33_NO_5_, [M + Na]^+^, 438.2256. Found 438.2249.

(1R,2S,4S)-1,7,7-trimethylbicyclo[2.2.1]heptan-2-yl (2-(4-formylphenoxy)acetyl) leucinate(**TB-4**): 73% yield; ^1^H NMR (400 MHz, CDCl_3_) δ 9.92 (s, 1H), 7.88 (d, *J* = 8.8 Hz, 2H), 7.07 (d, *J* = 8.7 Hz, 2H), 6.93 (d, *J* = 8.3 Hz, 1H), 4.92 (dd, *J* = 6.8 Hz, 2.1, 1H), 4.80-4.67 (m, 1H), 4.64-4.48 (m, 2H), 2.71-2.18 (m, 1H), 1.98-1.82 (m, 1H), 1.82-1.48 (m, 6H), 1.38-1.11 (m, 2H), 1.02 (dd, *J*_1_ = 13.9 Hz, *J*_2_ = 3.4 Hz, 1H), 0.96 (dd, *J*_1_ = 5.9 Hz, *J*_2_ = 3.9 Hz, 5H), 0.90 (s, 3H), 0.88 (s, 3H), 0.82 (s, 3H). ^13^C NMR (101 MHz, CDCl_3_) δ 190.6, 172.7, 166.8, 161.8, 132.1, 131.2, 115.1, 81.6, 77.3, 77.0, 76.7, 67.2, 50.7, 48.8, 47.9, 44.8, 42.0, 36.6, 27.9, 27.1, 24.9, 22.7, 22.1, 19.6, 18.8, 13.5. HRMS (ESI): Exact mass calcd for C_25_H_35_NO_5_, [M + Na]^+^, 452.2407. Found 452.2399.

(1R,2S,4S)-1,7,7-trimethylbicyclo[2.2.1]heptan-2-yl-N-(2-(4-formylphenoxy) acetyl)-O-methylserinate (**TB-5**): 79% yield; ^1^H NMR (400 MHz, CDCl_3_) δ 9.92 (d, *J* = 11.7 Hz, 1H), 7.87 (d, *J* = 8.8 Hz, 1H), 7.35 (d, *J* = 6.0 Hz, 1H), 7.07 (d, *J* = 8.6 Hz, 1H), 4.98 (m, 1H), 4.82 (dt, *J*_1_ = 8.0 Hz, *J*_2_ = 3.0 Hz, 1H), 4.61 (d, *J* = 1.9 Hz, 1H), 3.86 (dd, *J*_1_ = 9.4 Hz, *J*_1_ = 3.1 Hz, 1H), 3.69 (dt, *J*_1_ = 9.4 Hz, *J*_2_ = 3.1 Hz, 1H), 3.33 (d, *J* = 5.3 Hz, 1H), 2.45-2.26 (m, 1H), 2.47-2.25 (m, 1H), 1.95-1.81 (m, 1H), 1.80-1.61 (m, 1H), 1.39-1.11 (m, 1H), 1.07-0.93 (m, 1H), 0.90 (s, 1.5H), 0.89 (s, 1.5H), 0.87 (s, 3H), 0.84 (s, 1.5H), 0.82 (s, 1.5H); ^13^C NMR (101 MHz, CDCl_3_) δ 190.6, 169.8, 167.0, 161.8, 132.1, 131.1, 115.1, 81.9, 81.5, 77.3, 77.0, 76.7, 72.3, 72.27, 67.2, 59.3, 59.1, 52.6, 52.5, 49.1, 48.8, 47.9, 47.8, 44.8, 44.8, 36.7, 36.3, 29.7, 28.0, 27.9, 27.1, 19.7, 18.8, 13.4, 13.3; HRMS (ESI): Exact mass calcd for C_23_H_32_NO_6_, [M + NH]^+^, 418.2224. Found 418.2220.

(1R,2S,4S)-1,7,7-trimethylbicyclo[2.2.1]heptan-2-yl (2-(4-formylphenoxy)acetyl) phenylalaninate (**TB-6**): 74% yield; ^1^H NMR (400 MHz, CDCl_3_) δ 9.91 (s, 1H), 7.85 (dd, *J*_1_ = 8.9 Hz, *J*_2_ = 2.4 Hz 2H), 7.46-7.27 (m, 4H), 7.07-6.88 (m, 4H), 6.95-6.60 (m, 3H), 5.02 (s, 2H), 4.97-4.80 (m, 2H), 4.58-4.52 (m, 1H), 3.14-3.05 (m, 1H), 1.39-1.08 (m, 3H), 0.88 (s, 3H), 0.86 (s, 3H), 0.78 (s, 3H); ^13^C NMR (101 MHz, CDCl_3_) δ 190.6, 190.3, 171.3, 166.7, 166.3, 161.8, 160.6, 158.0, 157.00, 136.9, 132.1, 131.1, 130.3, 130.3, 129.6, 128.61, 128.00, 127.60, 127.42, 124.51, 115.04, 115.02, 99.99, 81.9, 80.7, 77.3, 77.0, 76.7, 71.5, 69.9, 68.4, 67.3, 61.1, 52.9, 52.5, 48.8, 48.8, 47.8, 46.9, 44.8, 43.4, 37.3, 36.6, 36.4, 34.9, 27.9, 27.0, 19.7, 19.6, 18.8, 16.5, 13.5, 13.3. HRMS (ESI): Exact mass calcd for C_28_H_23_NO_5_, [M + NH]^+^, 486.2256. Found 486.2251.

(1R,2S,4S)-1,7,7-trimethylbicyclo[2.2.1]heptan-2-yl-3-(4-(benzyloxy)phenyl)-2-(2-(4- formyl- phenoxy)acetamido)propanoate (**TB-7**): 72% yield; ^1^H NMR (400 MHz, CDCl_3_) δ 9.91 (s, 1H), 7.85 (dd, *J*_1_ = 8.9 Hz, *J*_2_ = 2.4 Hz, 2H), 7.46-7.27 (m, 4H), 7.07-6.88 (m, 4H), 6.95-6.60 (m, 3H), 5.02 (s, 2H), 4.97-4.80 (m, 2H), 4.58-4.52 (m, 1H), 3.14-3.05 (m, 1H), 1.39-1.08 (m, 3H), 0.89 (s, 2H), 0.88 (s, 1H), 0.87 (s, 2H), 0.86 (d, 1H), 0.79 (s, 2H), 0.77 (s, 1H);^13^C NMR (101 MHz, CDCl_3_) δ 190.6, 171.3, 166.7, 161.8, 158.0, 136.9, 132.1, 131.1, 130.3, 130.2, 128.6, 128.0, 127.6, 127.4, 115.0, 115.0, 99.9, 81.9, 69.9, 67.2, 52.9, 48.8, 47.8, 44.8, 37.4, 36.6, 27.9, 27.0, 19.6, 18.8, 13.5; HRMS (ESI): Exact mass calcd for C_35_H_39_NO_6_, [M + Na]^+^, 596.2635. Found 596.2660.

(1R,2S,4S)-1,7,7-trimethylbicyclo[2.2.1]heptan-2-yl (2-(4-(hydroxymethyl) phenoxy)acetyl)tyrosinate (**TB-8**): 75% yield; ^1^H NMR (400 MHz, CDCl_3_) δ 7.34-7.06 (m, 1H), 6.93-6.76 (m, 2H), 6.73 (t, *J*_1_ = 7.9 Hz, 1H), 6.54 (t, *J*_1_ = 7.2 Hz, 1H), 4.99-4.86 (m, 1H), 4.82 (dd, *J*_1_ = 13.2 Hz, *J*_2_ = 7.1 Hz, 1H), 4.64 (s, 1H), 4.44 (dt, *J*_1_ = 15.6 Hz, *J*_2_ = 10.5 Hz, 1H), 3.20-3.02 (m, 1H), 2.94 (td, *J*_1_ = 14.5 Hz, *J*_2_ = 7.0 Hz, 1H), 2.74 (s, 1H), 2.36 (m, 1H), 1.92-.81 (m, 1H), 1.81-.63 (m, 1H), 1.40-1.12 (m, 1H), 1.03-0.97 (m, 1H), 0.90 (s, 2H), 0.89 (s, 1H), 0.87 (s, 3H), 0.84 (s, 2H), 0.81 (s, 1H); ^13^C NMR (101 MHz, CDCl_3_) δ 171.5, 168.5, 168.4, 156.8, 155.1, 133.8, 130.1, 130.0, 129.4, 129.3, 126.5, 115.7, 114.5, 81.8, 77.3, 77.0, 76.7, 67.2, 64.9, 53.1, 53.0, 48.9, 48.8, 47.9, 47.8, 44.8, 36.9, 36.5, 36.5, 27.9, 27.9, 27.1, 19.6, 18.8, 13.6, 13.4; HRMS (ESI): Exact mass calcd for C_28_H_35_NO_6_, [M + H]^+^, 482.2543. Found 482.2528.

(1R,2S,4S)-1,7,7-trimethylbicyclo[2.2.1]heptan-2-yl-2-(2-(4-formylphenoxy) acetamido)-3-methylpentanoate (**TB-9**): 73% yield; ^1^H NMR (400 MHz, CDCl_3_) δ 9.92 (s, 1H), 7.88 (d, *J* = 8.7 Hz, 1H), 7.16-6.93 (m, 1H), 5.02-4.82 (m, 1H), 4.71 (dd, *J*_1_ = 8.7 Hz, *J*_2_ = 4.5 Hz, 1H), 4.67-4.42 (m, 1H), 2.43-2.21 (m, 1H), 2.03 -.83 (m, 1H), 1.81-1.65 (m, 1H), 1.53-1.37 (m, 1H), 1.37-1.11 (m, 1H), 1.01 (dd, *J*_1_ = 13.9 Hz, *J*_2_ = 3.3 Hz, 1H), 0.93 (dd, *J* = 9.8 Hz, 4.9 Hz, 1H), 0.90 (s, 3H), 0.88 (s, 3H), 0.83 (s, 3H); ^13^C NMR (101 MHz, CDCl_3_) δ 190.6, 171.5, 166.9, 161.8, 132.1, 131.2, 115.1, 81.8, 77.3, 77.0, 76.7, 67.3, 56.3, 48.78, 47.9, 44.8, 38.2, 36.7, 27.9, 27.1, 25.2, 19.6, 18.8, 15.5, 13.5, 11.6; HRMS (ESI): Exact mass calcd for C_25_H_35_NO_6_ [M + H]^+^, 430.2888. Found 430.2583.

1,7,7-trimethylbicyclo[2.2.1]heptan-2-yl (2-(4-formylphenoxy)acetyl)prolinate (**TB-10**): 76% yield; ^1^H NMR (400 MHz, CDCl_3_) δ 9.88 (s, 1H), 8.00-7.63 (m, 1H), 7.03 (t, *J* = 9.1 Hz, 1H), 5.04-4.51 (m, 1H), 3.81-3.47 (m, 1H), 2.47-2.25 (m, 1H), 2.19 (dd, *J*_1_ = 13.9 Hz, *J*_2_ = 5.6 Hz, 1H), 2.14-1.95 (m, 1H), 1.95-1.80 (m, 1H), 1.80-1.60 (m, 1H), 1.36-1.18 (m, 1H), 1.09 (dd, *J*_1_ = 34.2 Hz, *J*_2_ = 13.5 Hz, 1H), 0.88 (s, 3H), 0.85 (s, 3H), 0.79 (s, 3H), 0.76 (s, 1H); ^13^C NMR (101 MHz, CDCl_3_) δ 190.7, 190.7, 172.0, 171.9, 166.2, 165.8, 162.9, 162.6, 132.0, 130.8, 130.6, 115.7, 114.9, 81.5, 80.8, 77.4, 77.0, 76.73, 68.0, 67.3, 59.5, 59.2, 48.9, 47.9, 47.9, 47.4, 46.3, 44.8, 44.7, 36.7, 36.4, 32.0, 28.9, 28.0, 27.9, 27.1, 24.8, 21.9, 19.6, 19.6, 18.8, 18.7, 13.5; HRMS (ESI): Exact mass calcd for C_24_H_31_NO_5_ [M + H]^+^, 414.2275. Found 414.2268.

(1R,2S,4S)-1,7,7-trimethylbicyclo[2.2.1]heptan-2-yl-N-(2-(4-formylphenoxy) acetyl)-S-methylcysteinate (**TB-11**): 73% yield; ^1^H NMR (400 MHz, CDCl_3_) δ 9.92 (s, 1H), 7.89 (d, *J* = 8.7 Hz, 1H), 7.28 (s, 1H), 7.07 (d, *J* = 8.6 Hz, 1H), 5.02-4.88 (m, 1H), 4.90-4.76 (m, 1H), 4.69-4.46 (m, 1H), 2.51 (t, *J* = 7.2 Hz, 1H), 2.43-2.30 (m, 1H), 2.22 (dt, *J*_1_ = 12.8 Hz, *J*_2_ = 7.3 Hz, 1H), 2.13-2.01 (m, 2H), 1.92-1.83 (m, 1H), 1.82-1.66 (m, 1H), 1.40-1.12 (m, 2H), 0.99 (m, 1H), 0.91 (s, 3H), 0.88 (s, 3H), 0.85 (s, 1.5H), 0.83 (s, 1.5H); ^13^C NMR (101 MHz, CDCl_3_) δ 190.6, 183.7, 171.6, 171.5, 171.5, 171.3, 167.0, 164.5, 161.7, 143.5, 132.1, 131.5, 131.2, 130.3, 115.0, 103.2, 81.9, 81.1, 67.2, 54.8, 51.6, 51.6, 51.4, 51.2, 48.9, 48.1, 47.9, 45.0, 44.8, 44.8, 41.8, 36.8, 36.7, 36.5, 35.42, 31.8, 31.8, 31.6, 31.5, 29.9, 29.8, 29.7, 29.7, 28.6, 28.0, 27.9, 27.4, 27.1, 21.0, 19.6, 19.1, 18.8, 18.2, 15.5, 15.4, 13.6, 13.6, 13.5; HRMS (ESI): Exact mass calcd for C_23_H_31_NO_5_S [M + H]^+^, 434.1996. Found 437.1991.

(1R,2S,4S)-1,7,7-trimethylbicyclo[2.2.1]heptan-2-yl-2-(2-(4-formylphenoxy) acetamido)pentanoate (**TB-12**): 78% yield; ^1^H NMR (400 MHz, CDCl_3_) δ 9.92 (s, 1H), 7.88 (d, *J* = 8.6 Hz, 2H), 7.07 (d, *J* = 8.5 Hz, 3H), 4.94 (d, *J* = 9.6 Hz, 1H), 4.73 (dd, *J*_1_ = 13.1 Hz, *J*_2_ = 7.2 Hz, 1H), 4.60 (d, *J* = 3.0 Hz, 2H), 2.45-2.26 (m, 1H), 1.96-1.82 (m, 2H), 1.82-1.65 (m, 3H), 1.28 (m, 5H), 1.01 (dt, *J*_1_ = 10.5 Hz, *J*_2_ = 5.2 Hz, 1H), 0.90 (s, 3H), 0.88 (s, 3H), 0.82 (s, 3H); ^13^C NMR (101 MHz, CDCl_3_) δ 190.6, 172.3 166.8, 161.8, 132.1, 131.1, 115.1, 81.6, 67.2, 52.0, 48.9, 47.9, 44.8, 36.5, 34.7, 27.9, 27.1, 19.7, 18.8, 18.5, 13.7, 13.5; HRMS (ESI): Exact mass calcd for C_24_H_33_NO_5_ [M + H]^+^, 438.2251. Found 438.2245.

(1R,2S,4S)-1,7,7-trimethylbicyclo[2.2.1]heptan-2-yl (2-(4-formylphenoxy) acetyl)-L-tryptophanate (**TB-13**): 77% yield; ^1^H NMR (400 MHz, CDCl_3_) δ 9.88 (s, 5H), 8.05 (s, 5H), 7.77 (d, *J* = 8.8 Hz, 10H), 7.56 (d, *J* = 7.8 Hz, 6H), 7.35 (d, *J* = 8.1 Hz, 7H), 7.19 (t, *J* = 7.2 Hz, 6H), 7.07 (t, *J* = 7.5 Hz, 5H), 7.01 (d, *J* = 8.2 Hz, 5H), 6.80 (d, *J* = 8.7 Hz, 8H), 5.07-4.99 (m, 5H), 4.86 (d, *J* = 9.1 Hz, 5H), 4.59-4.46 (m, 9H), 3.37 (qd, *J*_1_ = 14.9 Hz, *J*_2_ = 5.8 Hz, 10H), 2.36-2.26 (m, 5H), 1.83-1.62 (m, 16H), 1.25 (dd, *J*_1_ = 15.6 Hz, *J*_2_ = 9.5 Hz, 11H), 1.16-1.08 (m, 5H), 0.88 (s, 2.5H), 0.87 (s, 0.5H), 0.85 (s, 2.5H), 0.83 (s, 0.5H), 0.77 (s, 2.5H), 0.70 (s, 0.3H); ^13^C NMR (101 MHz, CDCl_3_) δ = 190.6, 182.8, 171.6, 168.5, 166.9, 166.3, 161.7, 153.6, 136.1, 135.0, 131.9, 131.6, 130.9, 130.1, 122.6, 122.5, 122.4, 120.3, 119.9, 118.8, 118.6, 116.1, 114.9, 114.1, 111.3, 110.5, 109.8, 89.4, 81.7, 80.8, 69.6, 67.2, 53.8, 52.9, 52.5, 48.8, 48.3, 47.8, 46.8, 44.8, 39.3, 36.6, 31.5, 27.8, 27.3, 27.0, 24.3, 19.6, 19.1, 18.8, 16.8, 13.5; HRMS (ESI): Exact mass calcd for C_24_H_33_NO_5_ [M + H]^+^, 503.2540. Found 503.2571.

(1R,2S,4S)-1,7,7-trimethylbicyclo[2.2.1]heptan-2-yl-N6-acetyl-N2-(2-(4-formylphenoxy)acetyl)-L-lysinate (**TB-14**): 79% yield; ^1^H NMR (400 MHz, CDCl_3_) δ 9.93 (s, 1H), 7.89 (d, *J* = 8.8 Hz, 2H), 7.14 (d, *J* = 8.2 Hz, 1H), 7.09 (d, *J* = 8.7 Hz, 2H), 5.60 (s, 1H), 4.96 (d, *J* = 9.1 Hz, 1H), 4.71 (td, *J*_1_ = 7.9 Hz, *J*_2_ = 5.1 Hz, 1H), 4.66-4.50 (m, 2H), 3.21 (dd, *J*_1_ = 12.7 Hz, *J*_2_ = 5.8 Hz, 2H), 2.36 (d, *J* = 3.8 Hz, 1H), 1.96 (s, 2H), 1.80 (m, 6H), 1.54 (dt, *J*_1_ = 13.4 Hz, *J*_2_ = 6.6 Hz, 2H), 1.42-1.15 (m, 4H), 1.01 (dd, *J*_1_ = 13.9 Hz, *J*_2_ = 3.4 Hz, 1H), 0.91 (s, 3H), 0.88 (s, 3H), 0.82 (s, 3H); ^13^C NMR (101 MHz, CDCl_3_) δ 190.7, 184.3, 172.0, 170.9, 170.2, 168.7, 167.1, 162.9, 161.7, 132.1, 131.5, 131.2, 121.3, 115.1, 112.9, 81.7, 71.9, 67.2, 57.6, 51.8, 50.9, 48.9, 47.9, 46.9, 44.8, 40.1, 39.2, 38.2, 36.5, 33.6, 32.5, 30.0, 28.9, 28.2, 27.9, 27.1, 26.9, 23.3, 22.5, 20.7, 19.6, 19.0, 18.8, 17.5, 13.6; HRMS (ESI): Exact mass calcd for C_27_H_38_N_2_O_6_ [M + H]^+^, 487.2803. Found 487.2810.

1,7,7-trimethylbicyclo[2.2.1]heptan-2-yl-1-(2-(4-formylphenoxy)acetyl) piperidine-2-carboxylate (**TB-15**): 75% yield; ^1^H NMR (400 MHz, CDCl_3_) δ 9.87 (s, 1H), 7.82 (d, *J* = 8.7 Hz, 1H), 7.04 (d, *J* = 8.7 Hz, 2H), 5.30 (d, *J* = 5.1 Hz, 1H), 5.04-4.60 (m, 4H), 3.77 (d, *J* = 12.3 Hz, 1H), 3.34 (t, *J* = 12.3 Hz, 1H), 2.39-2.28 (m, 4H), 1.77-1.64 (m, 8H), 1.50-1.19 (m, 7H), 0.89 (d, *J* = 1.7 Hz, 2H), 0.86 (s, 3H), 0.82 (s, 1.5H), 0.80 (s, 2H); ^13^C NMR (101 MHz, CDCl_3_) δ 190.7, 170.9, 170.9, 166.9, 163.0, 131.9, 130.7, 130.6, 115.0, 81.2, 81.0, 66.9, 52.6, 52.5, 48.9, 48.8, 47.8, 47.8, 44.8, 44.8, 43.0, 43.01, 37.0, 36.6, 28.0, 27.2, 26.7, 26.5, 25.2, 20.9, 20.8, 19.7, 18.8, 13.6, 13.6. HRMS (ESI): Exact mass calcd for C_25_H_33_NO_5_ [M + H]^+^, 428.2431. Found 428.2432.

(1R,4S)-1,7,7-trimethylbicyclo[2.2.1]heptan-2-yl-3-(2-(4-formylphenoxy) acetamido)propanoate (**TB-16**): 79% yield; ^1^H NMR (400 MHz, CDCl_3_) δ 9.90 (s, 1H), 7.85 (d, *J* = 8.7 Hz, 1H), 7.11 (s, 1H), 7.02 (d, *J* = 8.7 Hz, 1H), 5.01-4.74 (m, 1H), 4.55 (s, 1H), 3.63 (dd, *J* = 12.1 Hz, 6.1, 1H), 2.59 (t, *J* = 6.0 Hz, 1H), 2.39-2.17 (m, 1H), 1.95-1.78 (m, 1H), 1.78-1.68 (m, 1H), 1.66 (t, *J* = 4.4 Hz, 1H), 1.39-1.08 (m, 1H), 0.88 (s, 1H), 0.85 (s, 1H), 0.79 (s, 1H); ^13^C NMR (101 MHz, CDCl_3_) δ 190.5, 172.5, 167.1, 161.8, 132.1, 131.1, 115.0, 80.6, 67.3, 48.8, 47.8, 44.8, 36.7, 34.6, 34.1, 27.9, 27.1, 19.7, 18.8, 13.5; HRMS (ESI): Exact mass calcd for C_22_H_29_NO_5_ [M + H]^+^, 410.1943. Found 410.1942.

**FIGURE 2:**
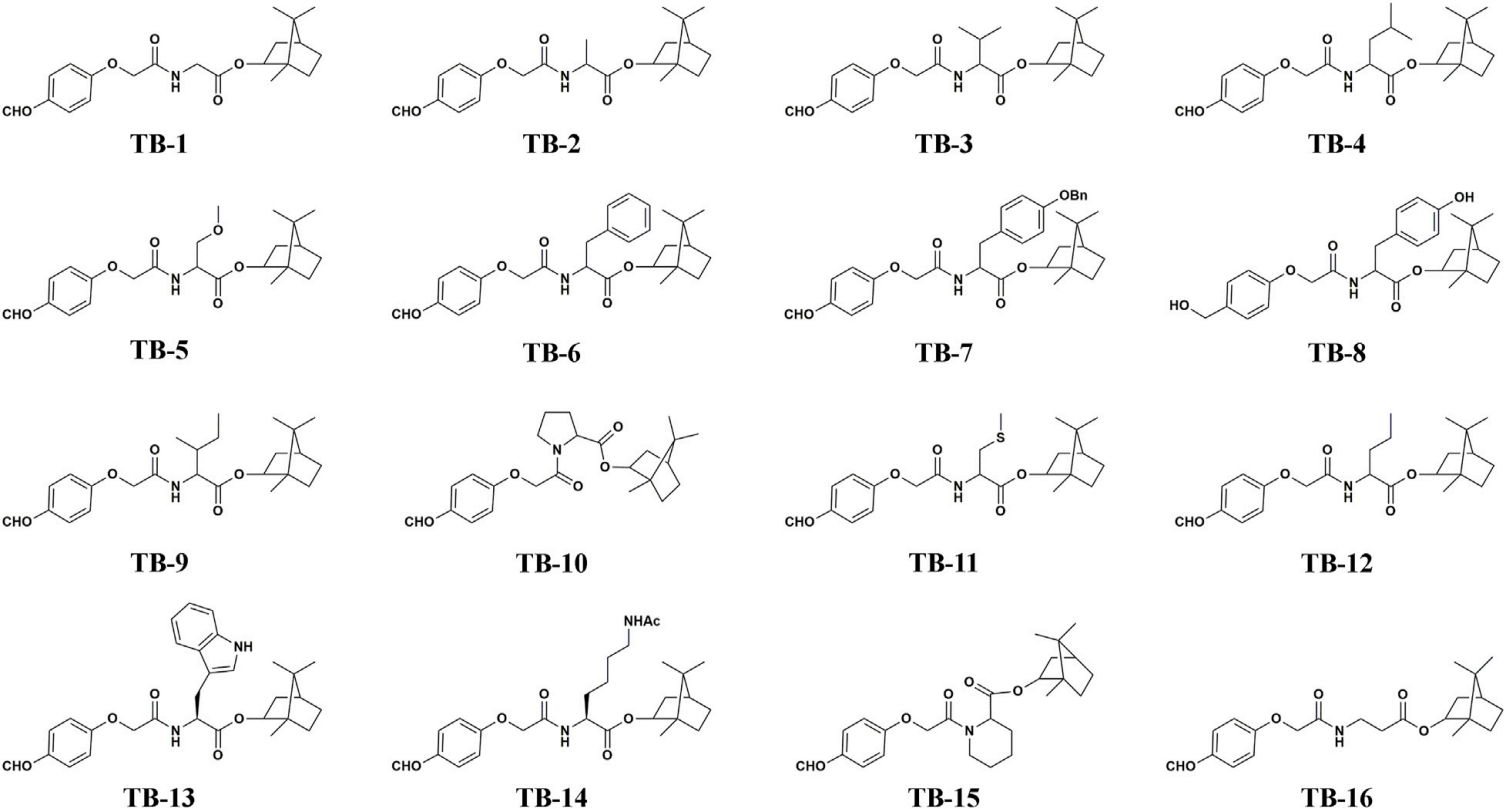
Structure of TB-1 to TB-16.

The structure of the compounds is shown in Figure 2.

### 3.2 The safety of TB-1∼TB-16

Prior to evaluating the protective efficacy of TB-1∼TB-16 against Glu injury, the safer optimal concentrations of these compounds must be ascertained. Subsequently, PC12 cells were exposed to varying concentrations of TB-1 to TB-16 (0, 0.1, 1, 10, 25, 50, 100, and 250 μM) for 24 h, and their cell viability was assessed using a CCK-8 assay. As indicated in Table 1, no significant reduction in cell survival rate was observed at low doses, whereas a dose-dependent decrease was evident with increasing compound dosage. *In vitro* experiments revealed that each compound exhibited a distinct safe concentration range. To further investigate their neuroprotective potential, we intend to select compounds with a wider safe concentration range for subsequent experiments. Since the cell survival rate exceeded 95% at an experimental concentration of 100 μM for TB-1, TB-2, TB-12, and TB-16, these compounds were chosen for subsequent neuroprotective experiments. The subsequent experimental concentrations were established as 0.1, 1, 10, and 100 μM.

**TABLE 1:** Safety assessment of TB-1 to TB-16.

Compound	Cell viability							
	0 μM	0.1 μM	1 μM	10 μM	25 μM	50 μM	100 μM	250 μM
TB-1	99.97 ± 0.95	98.07 ± 2.41	98.87 ± 0.34	100.64 ± 1.06	103.96 ± 0.97	104.63 ± 3.94	98.10 ± 2.04	39.58 ± 2.48
TB-2	100.03 ± 0.45	97.31 ± 1.87	99.46 ± 2.92	100.10 ± 3.21	102.12 ± 0.62	104.09 ± 2.70	107.13 ± 2.44	72.94 ± 3.01
TB-3	100.02 ± 3.57	97.39 ± 3.01	95.30 ± 4.51	95.33 ± 3.10	97.69 ± 3.34	95.64 ± 4.60	64.98 ± 3.22	7.09 ± 1.54
TB-4	100.00 ± 0.53	98.67 ± 2.32	97.31 ± 1.65	97.61 ± 4.21	99.24 ± 1.92	91.79 ± 4.16	39.45 ± 3.09	4.75 ± 0.47
TB-5	100.00 ± 0.53	98.54 ± 2.88	98.64 ± 2.59	97.34 ± 4.47	100.93 ± 2.28	103.16 ± 6.00	94.75 ± 5.74	67.03 ± 2.75
TB-6	100.03 ± 0.45	99.97 ± 3.61	99.78 ± 0.43	101.27 ± 3.77	91.41 ± 3.01	51.74 ± 0.62	15.95 ± 0.36	4.52 ± 0.21
TB-7	100.02 ± 1.81	97.46 ± 2.59	98.48 ± 3.24	98.50 ± 2.59	81.37 ± 12.46	68.82 ± 3.08	67.71 ± 4.43	72.45 ± 0.55
TB-8	99.98 ± 1.13	96.22 ± 0.55	97.96 ± 1.21	95.69 ± 1.74	89.16 ± 0.28	67.42 ± 2.25	31.25 ± 8.21	2.27 ± 0.24
TB-9	99.97 ± 9.87	103.85 ± 10.33	99.26 ± 10.53	102.63 ± 11.71	96.58 ± 6.66	90.03 ± 6.74	57.76 ± 7.38	0.80 ± 0.43
TB-10	100.00 ± 6.62	102.05 ± 2.59	102.51 ± 5.04	110.52 ± 6.37	108.76 ± 5.38	102.15 ± 7.22	42.36 ± 4.69	2.05 ± 0.65
TB-11	100.03 ± 2.14	99.62 ± 0.64	99.15 ± 2.78	103.40 ± 2.10	102.75 ± 1.16	95.90 ± 1.85	80.03 ± 2.04	70.60 ± 1.65
TB-12	100.03 ± 2.07	99.23 ± 0.72	99.31 ± 1.37	97.73 ± 3.05	98.00 ± 7.18	101.57 ± 3.26	100.08 ± 3.90	103.44 ± 2.37
TB-13	100.00 ± 1.35	98.32 ± 1.01	95.85 ± 2.61	86.66 ± 0.72	77.04 ± 1.54	72.16 ± 2.94	71.53 ± 0.99	44.37 ± 1.79
TB-14	99.98 ± 1.83	96.80 ± 4.96	96.83 ± 10.57	86.62 ± 8.03	96.14 ± 8.12	94.55 ± 8.84	68.05 ± 7.43	24.32 ± 1.95
TB-15	100.02 ± 7.36	101.11 ± 6.47	103.41 ± 8.53	106.84 ± 6.85	109.93 ± 10.67	68.19 ± 6.39	30.69 ± 3.29	5.25 ± 0.76
TB-16	99.97 ± 4.50	101.21 ± 3.31	99.70 ± 3.90	102.99 ± 6.37	104.94 ± 4.51	109.39 ± 5.61	99.78 ± 6.87	84.84 ± 6.12

### 3.3 Glutamate caused cell damage in cultured PC12 cells

To establish a model of oxidative stress injury, Glu was employed to induce PC12 cell death in this study. The optimal concentration of Glu that caused a 40%–60% decrease in PC12 cell viability was determined using the CCK-8 assay.

**FIGURE 3:**
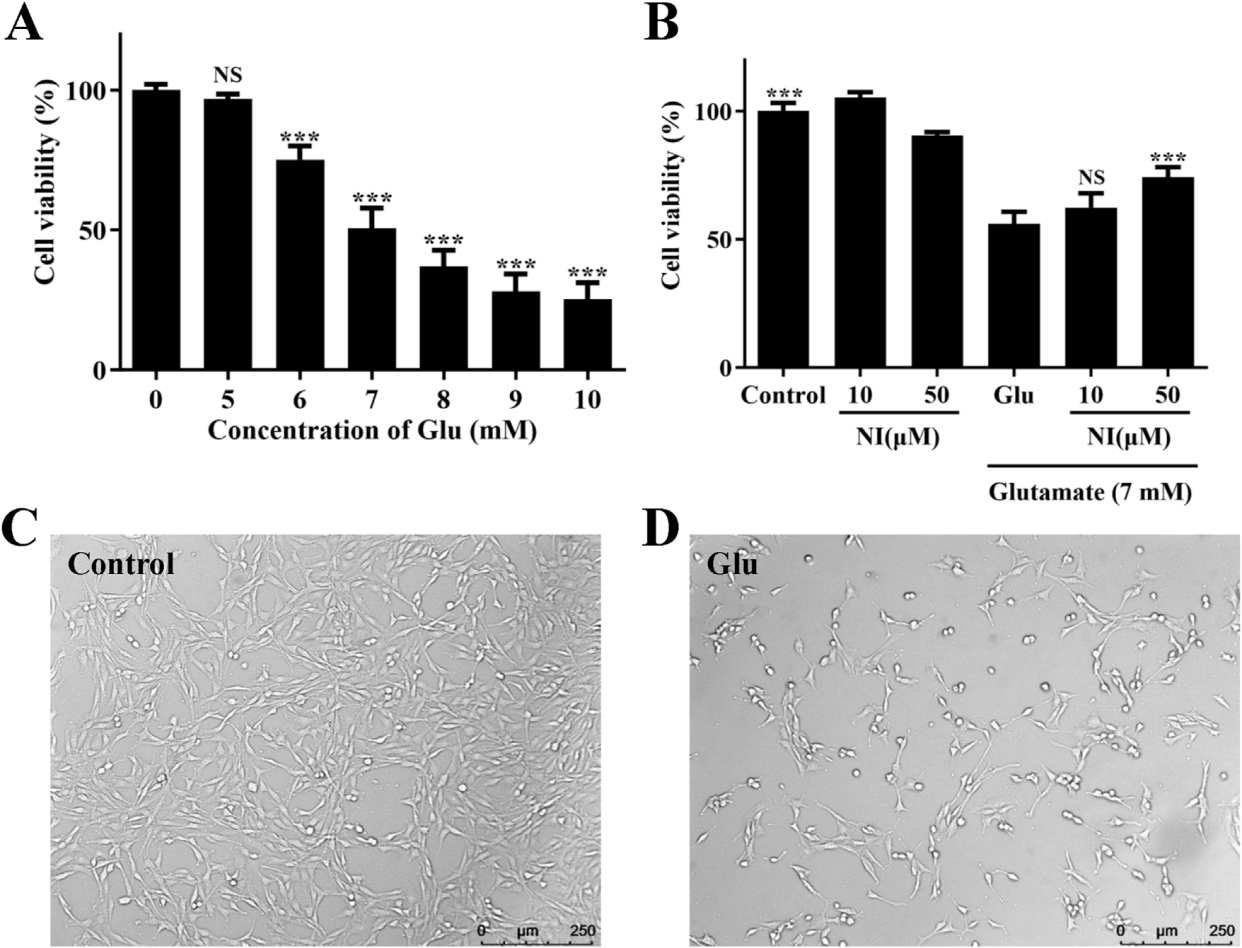
Glu-induced cell damage in cultured PC12 cells. **(A)** Effect of various Glu concentrations on PC12 cell viability, *p < 0.05, **p < 0.01, ***p < 0.001, vs. 0 group. **(B)** Protective effect of nimodipine (NI) pretreatment on Glu 7 mM-induced PC12 cell damage, ***p < 0.001, vs. Glu group. **(C)** Image of untreated PC12 cells. **(D)** PC12 cells after Glu treatment.

An *in vitro* model of neuronal injury was established in this study by exposing cultured PC12 cells to various concentrations (5, 6, 7, 8, 9, and 10 mM) of Glu for 24 h. The CCK-8 assay demonstrated that Glu significantly reduced cell viability in a concentration-dependent manner (Figure 3A). The survival rates of cells treated with glutamic acid at various concentrations (0, 5, 6, 7, 8, 9, and 10 mM) were 100.01% ± 2.18%, 96.93% ± 1.68%, 75.14% ± 4.91%, 50.56% ± 7.34%, 36.99% ± 5.72%, 28.00% ± 6.28%, and 25.15% ± 5.95%. Additionally, after exposure to 7 mM Glu for 24 h, cell viability was reduced to 50.56% ± 7.34%. Consequently, 7 mM Glu was utilized to induce neurotoxicity in the subsequent experiments.

The Glu injury model was validated using nimodipine. PC12 cells were pretreated with 10 and 50 μM nimodipine for 24 h, followed by exposure to 7 mM Glu for an additional 24 h. The CCK-8 assay revealed that, following 24-h treatment with 7 mM Glu, the survival rate of cells treated solely with Glu was 56.08% ± 4.69%. In contrast, cells pretreated with 10 and 50 μM nimodipine for 24 h exhibited survival rates of 62.37% ± 5.62% and 74.26% ± 3.84%, respectively (Figure 3B). Compared to control cells, cell viability was reduced to approximately 50% upon exposure to 7 mM Glu. However, pretreatment with 50 μM nimodipine significantly enhanced cell viability.

Following 24-h exposure to 7 mM Glu, microscopic observation revealed morphological changes in injured cells (Figures 3C, D). Control cells, which were not exposed to Glu, displayed a normal cell shape with an intact cell membrane, whereas Glu -exposed cells exhibited an early apoptotic morphology, characterized by plasma membrane blebbing and cell shrinkage. In summary, we have successfully established a Glu damage model, which can be utilized to validate the neuroprotective effect of the compound.

### 3.4 TB series compounds protects PC12 cells from glu-induced neurotoxicity

To investigate the neuroprotective effects of TB-1, TB-2, TB-12, and TB-16 against Glu excitotoxicity in PC12 cells, we examined the impact of varying compound types and concentrations on Glu-induced cell injury, utilizing the CCK-8 assay to assess cell viability.

**FIGURE 4:**
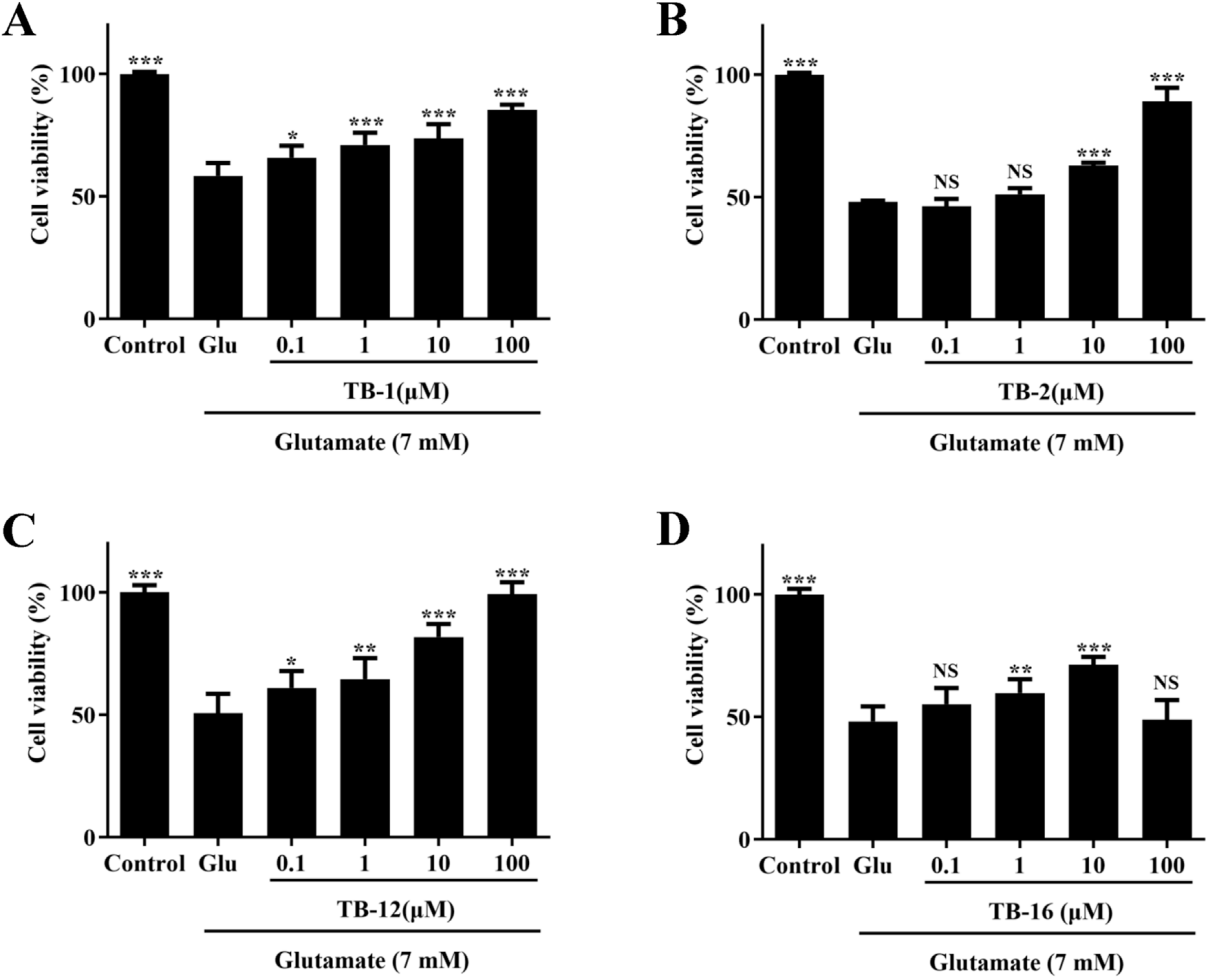
Effects of TB-1, TB-2, TB-12, and TB-16 on cell viability in Glu-stimulated PC12 cells. PC12 cells were treated with various concentrations of **(A)** TB-1 **(B)** TB-2 **(C)** TB-12 **(D)** TB-16 for 24 h, followed by 7 mM Glu for 24 h *p < 0.05, **p < 0.01, ***p < 0.001 vs. Glu group.

The cultured PC12 cells were pretreated with TB-1, TB-2, TB-12, and TB-16 at concentrations of 0.1, 1, 10, and 100 μM for 24 h, followed by exposure to 7 mM Glu for another 24 h. The CCK-8 assay revealed that, following 24-h treatment with 7 mM Glu, the viability of PC12 cells decreased to approximately 50% compared to control cells. However, pretreatment with TB-1, TB-2, TB-12, and TB-16 at concentrations of 0.1, 1, 10, and 100 μM increased cell viability to varying degrees (Figure 4). Pretreatment with TB-1 at concentrations of 0.1, 1, 10, and 100 μM increased cell survival rates by 7.39%, 12.53%, 15.29%, and 26.86%, respectively (Figure 4A). Pretreatment with TB-2 at concentrations of 10 and 100 μM resulted in observable increases in cell survival rates of 11.7% and 38.07%, respectively (Figure 4B). Pretreatment with TB-12 at concentrations of 0.1, 1, 10, and 100 μM led to observable increases in cell survival rates of 10.34%, 13.90%, 31.14%, and 48.76%, respectively (Figure 4C). Pretreatment with TB-16 at concentrations of 1 and 10 μM led to observable increases in cell survival rates of 11.59% and 23.04%, respectively (Figure 4D). However, at a concentration of 100 μM, TB-16 did not exhibit neuroprotective effects.

The findings indicate that TB-1, TB-2, TB-12, and TB-16 can attenuate the reduction in cell viability induced by Glu treatment, within a defined concentration range. At a concentration of 100 μM, TB-2 and TB-12 displayed notable neuroprotective effects, thereby enhancing the survival rate of Glu-damaged cells to 89.18% and 99.37%, respectively. Consequently, TB-2 and TB-12 were chosen for further network pharmacology and molecular docking analyses to elucidate their potential mechanisms of action in cerebral ischemia treatment and to furnish a theoretical basis and research orientation for subsequent experimental studies.

### 3.5 Molecular modeling study

**FIGURE 5:**
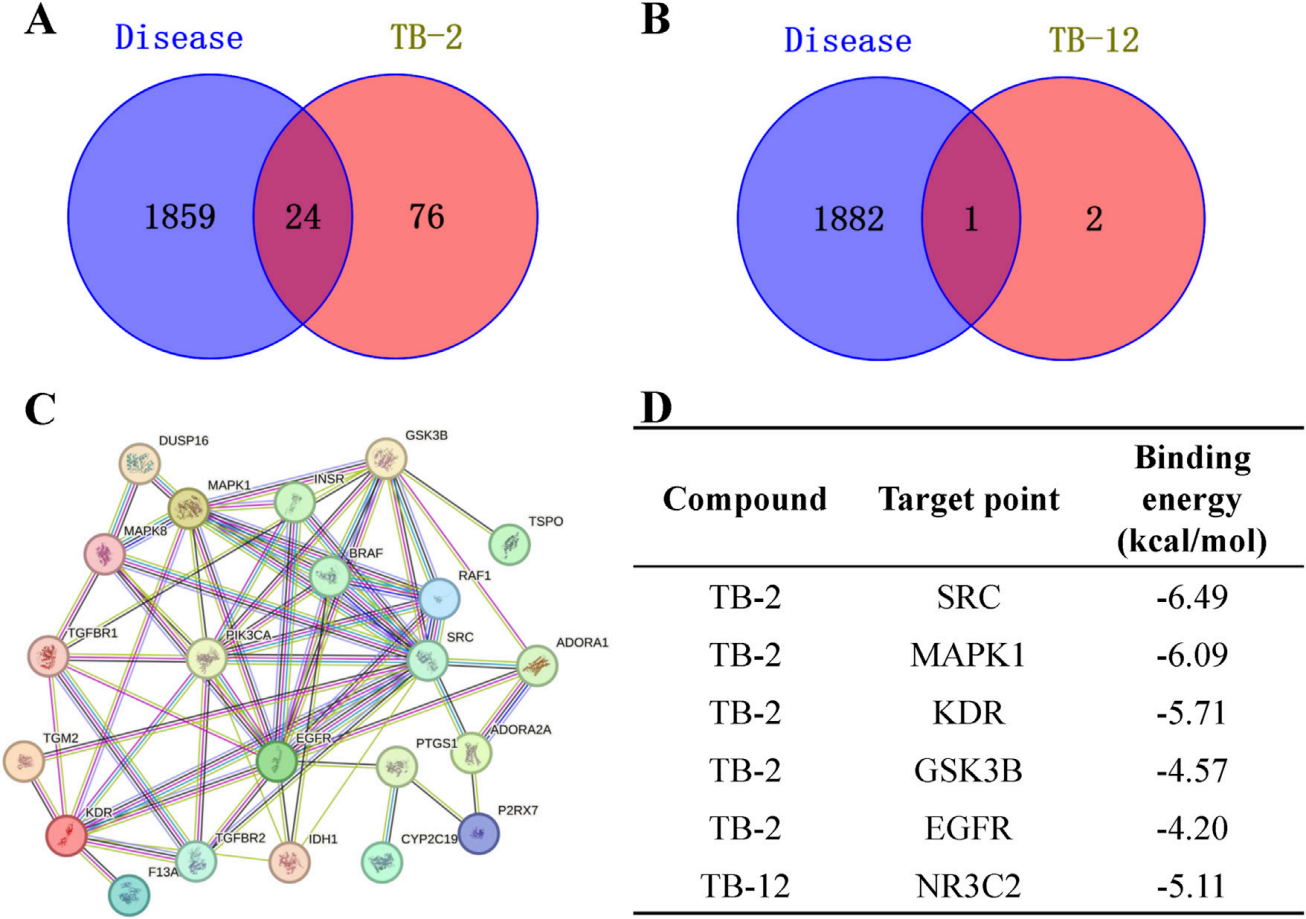
Network pharmacology analysis. **(A)** Wayne diagram of cerebral ischemia and TB-2 targets. **(B)** Wayne diagram of cerebral ischemia and TB-12 targets. **(C)** PPI network diagram of TB-2 in cerebral ischemia. **(D)** Molecular docking of core active ingredients with key targets.

Initially, 4283 cerebral ischemia targets were identified through the Genecards database. Following two rounds of screening employing the median method, 1760 targets were ultimately obtained. 107 cerebral ischemia targets were retrieved from the OMIM database. 120 cerebral ischemia targets were retrieved from the Disgenet database. Upon retrieval of cerebral ischemia targets from the Genecards, Disgenet, and OMIM databases, duplicates were eliminated, yielding a consolidated list of 1883 unique cerebral ischemia targets. Using SwissTargetPrediction, 100 TB-2 targets and 3 TB-12 targets were identified. Using the Venny 2.1 tool, 24 intersection targets of cerebral ischemia and TB-2, and 1 intersection target of cerebral ischemia and TB-12, were identified (Figures 5A, B). The intersection target between cerebral ischemia and TB-12 was NR3C2. The identified common targets of cerebral ischemia and TB-2 were imported into the STRING database for the construction of the PPI network of the intersection target protein (Figure 5C). The PPI network diagram comprised 22 nodes and 64 edges. Data was imported into Cytoscape 3.7.2 software for the acquisition of the PPI network of genipine’s target for cerebral ischemia treatment.The core targets of cerebral ischemia and TB-2 were screened based on the Betweenness (unDir), Closeness (unDir), and Degree values. When DC > 5.82, BC > 21.27, and CC > 0.02 were set as screening thresholds, five primary targets were identified: EGFR, SRC, GSK3B, MAPK1, and KDR.

**FIGURE 6:**
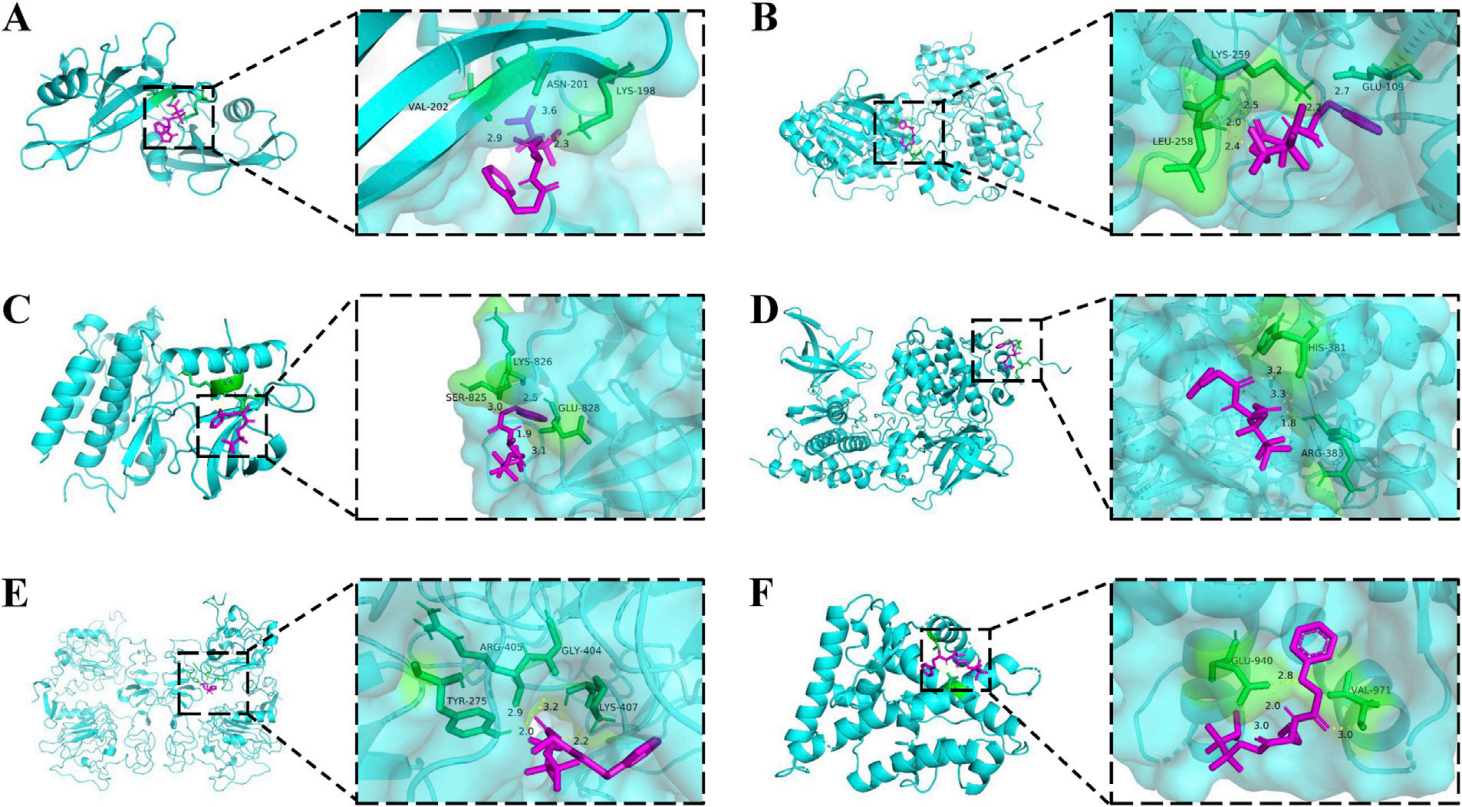
Molecular docking models of core active compounds and key targets. 2D (left) and 3D (right) representations of interactions between **(A)** TB-2 and SRC **(B)** TB-2 and MAPK1 **(C)** TB-2 and KDR **(D)** TB-2 and GSK3B **(E)** TB-2 and EGFR **(F)** TB-12 and NR3C2.

The core targets possessing the highest degree in the PPI network were selected for simulation docking with TB-2 and TB-12, respectively. The binding energies of TB-2 to SRC, MAPK1, KDR, GSK3B, and EGFR, in ascending order, were −6.49, −6.09, −5.71, −4.57, and −4.20 kcal/mol, respectively. The binding energies of TB-12 to NR3C2 was −5.11 (Figure 5D). The above findings were further validated by molecular docking schematics (Figure 6).

## 4 Discussion

The combination of Chinese medicine molecular chemistry strategies is founded on the design of innovative drug molecular entities in the clinical application of traditional Chinese medicine. This novel pharmaceutical design strategy involves selecting Chinese medicines, prescriptions, botanical medicines, and even the core effective molecules or metabolic products from chemical drugs. Consequently, we designed and synthesized a series of novel gastrodia-borneol compounds (TB-1∼TB-16). The neuroprotective activities of these compounds were evaluated on Glu-induced damage in PC12 cells, which are commonly utilized for screening novel candidates for stroke intervention via the CCK-8 assay. Furthermore, we delved deeper into the neuroprotective properties of TB-2 and TB-12, utilizing network pharmacology and molecular docking approaches, to elucidate the therapeutic mechanisms underlying their efficacy in cerebral ischemia.

PC12 cells exhibit ease of culture, unique synaptic formation ability, and neuro-associated protein production, making them a popular choice for constructing *in vitro* models of neurological diseases, such as stroke, Alzheimer’s disease, cerebral ischemia, and other disorders (Chua and Lim, 2021; Liu et al., 2021; Liu et al., 2023). In the current study, PC12 cells were exposed to Glu to establish an excitotoxic injury model. The effects of varying glutamate concentrations on PC12 cell viability were evaluated using cell viability assays. A Glu concentration of 7 mM was determined to be efficacious, similar to previous studies (Alavi et al., 2023).

We initially assessed the safe concentration range of compounds TB-1∼TB-16 by co-culturing them with PC12 cells, subsequently screening for the most optimal compounds for further investigation of their neuroprotective effects. The present study investigated whether TB-1, TB-2, TB-12, or TB-16 exhibited protective effects against Glu-induced neurotoxicity in PC12 cells. Among the tested TB molecules, TB-2 and TB-12 exhibited the most significant cytoprotective effect in the injury model, evidenced by reduced apoptotic rates. In summary, our findings indicate that TB-2 and TB-12 possesses a notable cytoprotective effect against Glu-induced injury in PC12 cells.

Stroke, a significant disease affecting human health, predominantly manifests as ischemic strokes. Despite advancements in pathological research, the effective regeneration of injured nerves remains challenging, thus necessitating the development of novel neuroprotective agents to mitigate ischemic brain injury (Wang et al., 2018). In network pharmacology studies, it has been found that TB-2 has more intersection targets with cerebral ischemia than TB-12. The core intersecting targets between cerebral ischemia and TB-2 are EGFR, SRC, GSK3B, MAPK1, and KDR, which may serve as potential therapeutic targets. The ligand’s binding affinity to the receptor is primarily assessed by the magnitude of the binding energy, where a smaller value indicates stronger binding ability and easier binding to the receptor (Siebenmorgen and Zacharias, 2019). Molecular docking analysis revealed lower binding energies of TB-2 with SRC, MAPK1, and KDR, and the significant role of these targets in cerebral ischemia treatment has been documented in previous literature (Mao et al., 2015; Liu et al., 2021; Chen et al., 2019). Utilizing SwissTargetPrediction, we identified 100 potential targets for TB-2. Beyond the previously mentioned EGFR, SRC, GSK3B, MAPK1, and KDR targets in our experimental findings, we further analyzed the remaining targets. Our results indicate that TB-2 may hold therapeutic potential not only in cerebral ischemia but also in cancer, neuroinflammation, neurodegenerative disorders, and metabolic diseases. Notably, majority targets of TB-2 are implicated in tumor therapy, including TGM2 (Zhang et al., 2023), KIT (Meng and Carvajal, 2019), EGFR (Zubair and Bandyopadhyay, 2023), AURKA (Du et al., 2021), FASN (Fhu and Ali, 2020), and CDK2 (Tadesse et al., 2020), which have garnered extensive research attention.

Collectively, our findings indicate that TB-2 exhibits a significant cytoprotective effect against Glu-induced injury in PC12 cells and holds potential therapeutic implications for cerebral ischemia. Further *in vivo* studies are necessary to validate the current findings. Additional studies are also required to elucidate the mechanism of action of TB-2 in neuroprotective function.

## Data Availability

The original contributions presented in the study are included in the article/supplementary material, further inquiries can be directed to the corresponding authors.
